# Time-Varying Pseudorandom Disturbed Pattern Generation Algorithm for Track Circuit Equipment Testing

**DOI:** 10.3390/mi13060853

**Published:** 2022-05-29

**Authors:** Xiaoming Chen, Zhixuan Wang, Zhiyang Yu, Hsiang-Chen Chui

**Affiliations:** 1 School of Optoelectronic Engineering and Instrumentation Science, Dalian University of Technology, Dalian 116000, China; chen_xm@dlut.edu.cn (X.C.); wangzhixuan2019@mail.dlut.edu.cn (Z.W.); 2 Signal and Communication Research Institute, China Academy of Railway Sciences Corporation Limited, Beijing 100081, China; gzmdyzy@126.com

**Keywords:** high-speed railway, ATPG 2, pseudorandom pattern 3, D algorithm 4

## Abstract

To improve the test accuracy and fault coverage of high-speed railway-related equipment boards, a time-varying pseudorandom disturbance algorithm based on the automatic test pattern generation technology in chip testing is proposed. The algorithm combines the pseudorandom pattern generation algorithm with the deterministic pattern generation D algorithm. The existing pseudorandom number generation method usually requires random seeds to generate a series of pseudorandom numbers. In this algorithm, the system timer is used as the random seed to design a pseudorandom pattern generation method of time-varying seed to improve the randomness of pseudorandom pattern generation. In addition, in combination with the D algorithm, this work proposes a new switching logic between two algorithms by counting invalid pattern proportions. When the algorithm is applied to track a circuit netlist, the fault coverage can reach near 100%. However, the large-scale circuit fault coverage cannot easily reach 100%. The test results for the standard circuits of different sizes show that at the same time, compared with the independent pattern generation methods, the proposed algorithm can improve fault coverage by more than 50% and 30% and significantly improve the pattern generation efficiency. Therefore, it can be used perfectly in the subsequent construction of high-speed railway equipment test platforms.

## 1. Introduction

Transportation is the lifeblood of the national economy and social development, and high-speed railways have been playing an important role in the comprehensive transportation system. The performance, safety testing, and verification of high-speed railway equipment are directly related to the safe operation of high-speed railway systems. Therefore, improving the test accuracy and fault coverage of related equipment boards and developing the corresponding test platform has become an urgent issue to be solved.

A test case is an effective solution to the traditional board test. CEDEX Interoperability Railways Laboratory established the Eurocab test platform for semi-automatic tests and realized the analysis and evaluation of European railway equipment through a test case [1]. Wu et al. proposed a test generation technique based on a colored Petri net model for an advanced satellite-based train control system to generate more complete test cases. The number of test cases increased by 23% after improving the model [2]. For the Chinese Train Control System 3, Zhou et al. optimized test cases through unified modeling language (UML) model diagrams using static and dynamic modeling mechanisms of the UML technology, effectively improving the generation efficiency and quality of cases [3]. Li et al. proposed the timed automata mutation test to simulate most types of system fault models, modeled the radio block center switching process, and improved the mutation score by approximately 11.8% through a mutation analysis. New test cases were generated to improve its integrity [4].

Although the aforementioned test case method has made great progress, it still needs to be improved in edge detection, system stability under limiting conditions, and test accuracy. The chip contains millions of logic gates. In its design process, many methods and EDA tools can verify its function, including automatic test pattern generation (ATPG) technology [5]. If the high-speed railway equipment or board is described as a circuit model, the method used in the integrated circuit design [6] thus had a reference significance to the system level test of the high-speed railway equipment.

The D algorithm is the first widely used deterministic test pattern generation algorithm in the ATPG technology. With the continuous improvement of chip complexity, the limitations of the D algorithm, which has a large number of backtracking and invalid choices, are constantly emerging [7]. Path-oriented decision-making (PODEM) is an improvement of the D algorithm, which reduces the backtracking times and blind trials of the D algorithm [8]. The fanout-oriented algorithm (FAN) is an improvement on PODEM, which significantly improves the generation efficiency by limiting the search space of ATPG to reduce the generation time and accelerate backtracking [9]. In addition, there are many other popular algorithms such as HITEC and Socrates. HITEC presented a targeted D element technique, which greatly increases the number of possible mandatory assignments and reduces the over-specification of state variables which can sometimes result when using a standard PODEM algorithm [10]. Socrates, based upon the sophisticated strategies of the FAN algorithm, led to a considerable reduction of the number of backtrackings and earlier recognition of conflicts and redundancies [11], but the generation process is still complicated.

Among the non-deterministic pattern generation methods for reducing the pattern generation time, pseudorandom pattern generation based on the linear feedback shift register (LFSR) is one of the most typical methods of creating a test pattern. It uses a pseudorandom number generator to generate test patterns and calculates the fault coverage of the generated patterns through fault simulations [12]. Compared with the deterministic pattern generation method, the pseudorandom generation method is simple, but it is more difficult to achieve a higher fault coverage. Moreover, the random seed of this method is single, and the randomness is limited. Souza et al. proposed a method combining the LFSR and deterministic generation algorithm [13]. However, there is still room for improvement in its randomness and generation efficiency.

Based on the combined generation algorithm, in this work, a time-varying pseudorandom disturbance ATPG algorithm, combining the pseudorandom pattern generation algorithm with the deterministic pattern generation D algorithm, is proposed. The existing pseudorandom number generation method usually requires random seeds to generate a series of pseudorandom numbers. In this algorithm, the system timer is used as the random seed to design a pseudorandom pattern generation method of time-varying seeds to improve the randomness of pseudorandom pattern generation. In addition, in combination with the D algorithm, here, a new switching logic between two algorithms by counting the invalid pattern proportion is proposed. The algorithm is applied to the transmitter and receiver board system of the ZPW-2000A track circuit, and the pattern generation of its golden model [14] circuit netlist is performed. Then, the efficiency of the algorithm is tested by comparing the circuits S713, S1423, and S9234.

## 2. Time-Varying Pseudorandom Disturbance ATPG Algorithm

The ATPG algorithm uses the gate-level netlist of a circuit as the input file and uses it to create a fault list to complete the pattern generation process. The fault list contains all possible fault types in the circuit, including the typical stuck-at fault, stuck-open fault, bridging fault, and some atypical faults. In this work, only the most common and effective stuck-at fault is considered, which represents the situation in which one line of the circuit is fixed to logic 1 or logic 0 and is represented as s-a-1 or s-a-0, respectively [15]. Netlist files are obtained through a logical synthesis of the register-transfer level code designed by Verilog.

### 2.1. A. Deterministic Pattern Generation Algorithm Based on the D Algorithm

**Figure 1:**
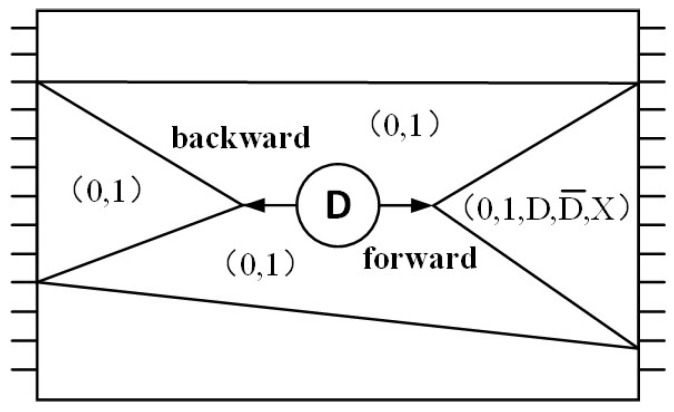
D algorithm schematic diagram.

The D algorithm is the most widely used deterministic pattern generation algorithm. It is based on path sensitization at the logic gate level [16] and uses a five-valued logic (0, 1, X, D, $\bar{D}$) to describe the states of each lead in the circuit under the failure condition. The principle is shown in Figure 1.

“0” indicates the signal with a normal value of 0 and fault value of 0. “1” indicates that the normal value is 1, and the fault value is also 1. “D” represents the signal with a normal value of 1 and fault value of 0, which can be recorded as 1/0. “$\bar{D}$” represents a signal with a normal value of 0 and fault value of 1, which can be recorded as 0/1. “X” indicates that the value is undetermined. The D algorithm is also implemented in C language and consists of three steps:

(a) Fault sensitization (backward): The influence of the fault can be reflected by driving the signal to be the opposite logical value of the fault. That is, activating the s-a-0 fault makes the input value of the line set to 1, whereas activating the s-a-1 fault makes the input value of the line set to 0. Thus, the logic value that the input can activate this fault is obtained through a further inverse calculation [17].
(b) Fault propagation (forward): The D signal is propagated to the output end of the circuit through one or more paths by setting the input node value of the relevant logic gates outside the fault point, so that it can be detected from the output end, and then inverse to improve the test pattern based on the hypothesis [18].
(c) Line confirmation: The undetermined signal value in the circuit is obtained using the existing node value until a set of non-contradictory values of the original input end of the circuit is obtained, which is a set of test patterns. When the selected value 0 or 1 is inconsistent with the previously assigned value of this node, it is necessary to go back to the previous node and select again [19].

Compared with a random generation, the D algorithm has a higher fault coverage, makes it easier to find undetectable faults, and uses fewer patterns. However, the calculation process is complex, requires more resources, and takes a long time to generate [20].

### 2.2. B. Time-Varying Seed Pseudorandom Pattern Generation Algorithm

**Figure 2:**
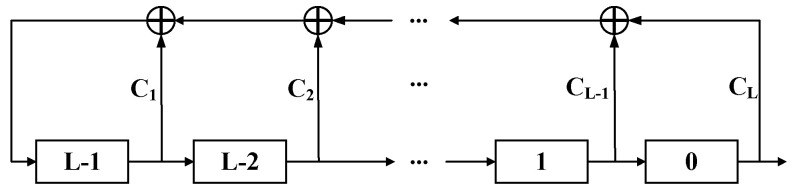
External XOR LFSR.

Pseudorandom pattern generation in integrated circuits is often used in a built-in self-test (BIST). As a common testability design method, it can significantly improve the testability of random logic in circuits. The BIST often uses LFSR to constitute a pseudorandom sequence generation circuit [21], which has the advantages of a simple generation process and high coverage in a short time. The external exclusive-OR (XOR) LFSR structure with a conventional length of L is shown in Figure 2.

In Figure 2, a set of initial assignment values between (L−1) and 0 is called a set of random seeds. At present, the quantum random number generators (QRNGs) have been used to achieve complete random-number generation [22], but it is too complicated for ATPG technology. The simpler pseudorandom number generation method usually requires random seeds to generate a series of fixed pseudorandom numbers [23]. The feedback constant *C_L_* is 0 or 1, and a single seed can generate a maximum of *2^L^* patterns. To generate other patterns, the random seed or feedback constant needs to be changed, and the randomness has a certain space for improvement. This work uses C language to realize the algorithm flow. The algorithm generates a pseudorandom sequence as a test pattern and then conducts fault simulation on each pattern to identify all faults that can be detected. After the detected faults are deleted from the fault list, the next test pattern is generated to repeat the process.

Because the random seed of the pseudorandom pattern generation method based on LFSR cannot be easily changed, the randomness needs to be improved. The algorithm uses the value of the system timer/counter to set the random seed so that the seed can change at any time and ensure that each pattern is generated by a different seed. By generating random numbers from 0 to 100 and judging whether they are greater than 50, 0/1 can be randomly generated by bits according to the required pattern length. Compared with LFSR in [13], the correlation between patterns is reduced, and the algorithm’s randomness is enhanced. The generating flow is as Algorithms 1:
**Algorithms 1:Pseudorandom pattern generation.**1: **if** seed < 1000 2:    **then** seed ← time(((long *) NULL)) 3:    **else** seed ← rand()% seed 4:        srand (seed) 5: **for** K = 0 to max do 6:    **if** rand()% 100 > 50 7:    **then** input[K] ← ‘1’ 8:    **else** input[K] ← ‘0’ 9: **end**

However, the generation of a pseudorandom pattern still has some problems, such as difficulty in finding undetectable faults, a long time to reach the target fault coverage, and a large number of required patterns, so the generation efficiency still has a large space to improve.

### 2.3. C. Time-Varying Pseudorandom Disturbance ATPG Algorithm

Based on the time advantage of the pseudorandom pattern generation algorithm and coverage advantage of the D algorithm, a time-varying pseudorandom disturbance ATPG algorithm is proposed in this work to improve the generation efficiency of the algorithm from two important evaluation indexes, i.e., fault coverage and generation time.

**Figure 3:**
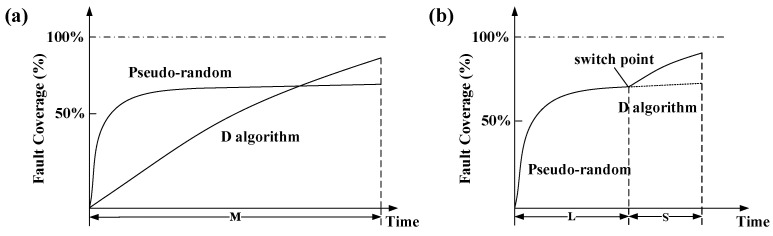
Relationship between the fault coverage and generation time of the algorithms. (**a**) with two independent algorithms, (**b**) with the time-varying pseudorandom disturbance algorithm.

Figure 3a shows the comparison of the variation trend of the fault coverage with the generation time between the pseudorandom generation algorithm and the D algorithm [24].

The early random patterns gradually slow down, which cannot be significantly improved even after a long time. However, the D algorithm always maintains a certain rate of coverage improvement, which is significantly higher than the generation of a pseudorandom pattern. This finding indicates that the algorithm can deal with many undetectable faults that cannot be found by the pseudorandom pattern.

Based on the generation characteristics of the two kinds of pattern generation methods, the time-varying pseudorandom disturbance ATPG algorithm is designed. The variation trend of the algorithm fault coverage with the generation time is shown in Figure 3b. The generation algorithm adopts the pseudorandom method first and then the D algorithm. First, the pseudorandom pattern generation is used to achieve a high fault coverage in 5 s. After the curve slows down, the D algorithm deals with the remaining undetectable faults in the fault list. Thus, considerable fault coverage can be achieved in a short time, and the generation efficiency can be significantly improved. Parameters *M*, *L,* and *S* represent the running time of the two algorithms. When the same coverage is achieved, the improved algorithm in Figure 3b can make the total generation time of the algorithm represented by *L + S* smaller than the time *M* in Figure 3a [13].

In the pseudorandom pattern generation process, when a generated pattern cannot detect any fault in the fault list, it is regarded as an invalid pattern. Variable *α* is used to count the invalid pattern ratio to achieve the control of the algorithm switching timing, adjust the ratio occupied by the two methods, and improve the generation efficiency. When *α* ≥ *n*, the generation of the pseudorandom pattern ends, and the D algorithm is used to continue the generation process. The specific control mode is shown as Algorithms 2:
**Algorithms 2:The control mode of the algorithm switching timing.**1: **while**
*α* < *n*
**do** 2:    Generate a random pattern 3:    Simulate the pattern 4:    **if** faults_count = 0 5:    **then**
*P*++ 6:     α←*P*/*Q* 7:    **else** Mark the fault as detected 8: **end** 9: Generate test patterns deterministically

**Figure 4:**
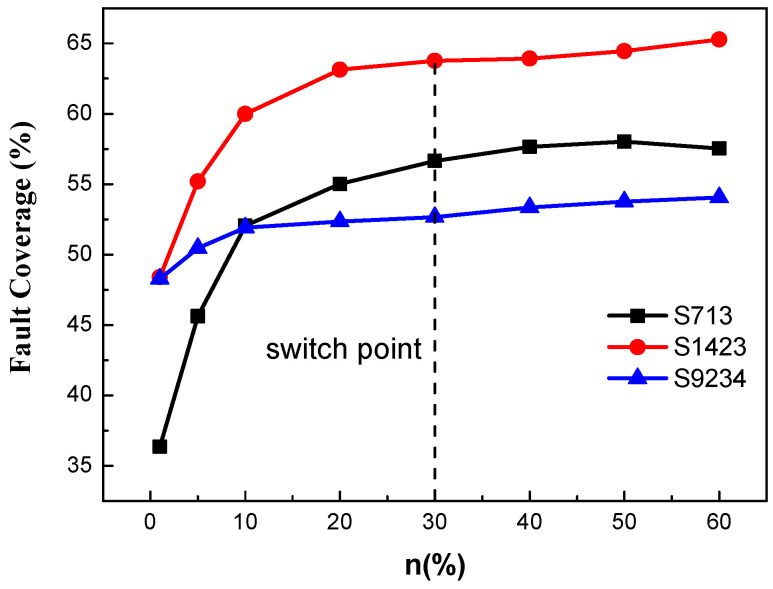
Pseudorandom patterns generate curve of the fault coverage varying with *n* and the algorithm switching point.

*P* represents the number of generated invalid patterns, and *Q* represents the total number of generated patterns. *α* is used as an invalid pattern ratio counter and represents the proportion of generated invalid patterns to the total number of generated patterns. Standard circuits S713, S1423, and S9234 are used for pseudorandom pattern generation, and the change in the fault coverage with *n* values is shown in Figure 4. It can be used to select the algorithm switching timing.

In this curve, it went smoothly and showed saturation, such that its derivative gradually approaches 0. For *n* = 10%, the rate of derivative decrease significantly slows down, and the fault coverage of pseudorandom patterns has basically reached the highest and does not change significantly with *n*. It is difficult to effectively improve the fault coverage by increasing the n value and has a limited influence on the overall coverage of the algorithm. To balance the fault coverage and generation time and ensure that the highest fault coverage can be achieved within the shortest time, *n* = 30% was selected as the algorithm switching point in this work. Compared with the switching logic of using timers to control the generation time of pseudorandom patterns adopted in [13], this work achieved a reasonable control of specific switching timing by using the same variable *n* in view of the actual pattern generation of different circuits. This method reduces blindness and makes the fault coverage of different circuits as consistent as possible at a certain switch point with the variation trend of variable *n*.

## 3. Results and Discussion

**Figure 5:**
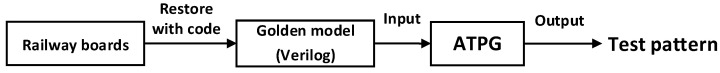
Algorithm test logic diagram.

The logic of the algorithm testing is described in Figure 5. The application of ATPG to high-speed railway equipment requires the design of a corresponding golden model. The golden model is designed according to the equipment system, and it can be used to obtain the netlist after a logical synthesis and inputted into the ATPG algorithm to generate patterns.

The transmitter and receiver of the high-speed railway ZPW-2000A track circuit were designed to realize the modulation and demodulation functions of frequency-shifted FSK signals, respectively.

**Figure 6:**
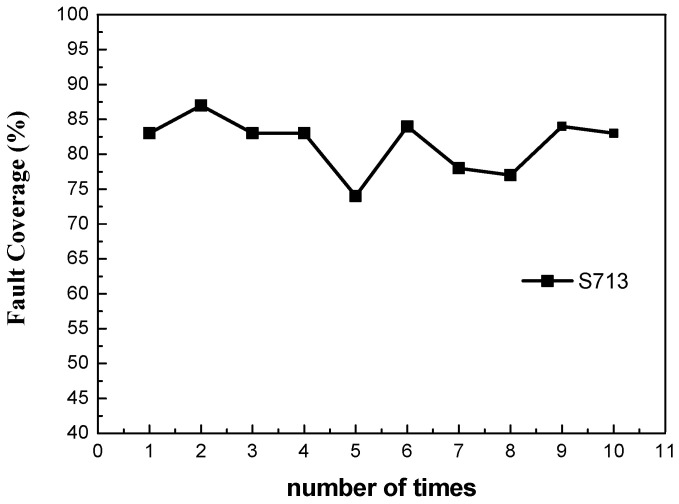
Results of multiple tests against the disturbance algorithm using S713.

First, the designed golden model netlist was used for the pattern generation (11 gates for the transmitter and 20 gates for the receiver), and the fault coverage can reach near 100% using the algorithm. As the large-scale circuit fault coverage cannot easily reach 100%, the medium-scale circuits S713 (393 gates) and S1423 (657 gates), and large-scale circuit S9234 (5597 gates) were tested. In the same base generation time, the algorithms using the pseudorandom pattern generation and D algorithm alone were compared with the disturbance algorithm. Due to the randomness in the algorithm, the fault coverage results generated by the algorithm are different each time. Take the multiple generation results of circuit S713 as an example, as shown in Figure 6.

**Figure 7:**
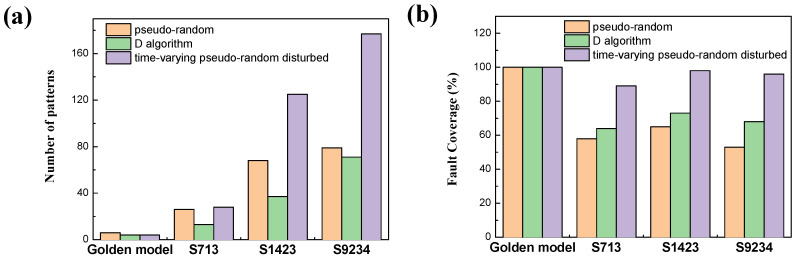
Generated results of each algorithm. (**a**) Number of patterns. (**b**) Fault coverage.

As can be seen from the figure, the results of multiple tests for the disturbance algorithm using S713 fluctuate within a certain range. Thus, five groups of repeated experiments were required to take the average value, and the number of generated patterns, detected faults, and fault coverage of each algorithm were counted, as shown in Table 1 and Figure 7.

The generation results in Figure 7 show that in the same generation time, compared with the D algorithm, the pseudorandom pattern generation has the lowest fault coverage and requires more patterns. The number of detected faults and fault coverage reached the highest level after the combination of the two methods. The fault coverage increased by more than 50% and 30% compared with the independent method, so the efficiency of the algorithm was significantly improved.

## 4. Conclusions

To improve the test coverage of high-speed railway-related equipment systems, a time-varying pseudorandom disturbance ATPG algorithm is proposed. The algorithm combines the pseudorandom pattern generation algorithm with the deterministic pattern generation D algorithm. In this algorithm, the system timer is used as the random seed to design a pseudorandom pattern generation method of time-varying seeds. It can improve the randomness of the pseudorandom pattern generation in [13]. In addition, in combination with the D algorithm, this work proposes a new switching logic between two algorithms by counting the invalid pattern proportion. Compared with the switching logic of using timers to control the generation time of pseudorandom patterns adopted in [13], this proposed method reduces blindness and makes the fault coverage of different circuits as consistent as possible at a certain switch point with the variation trend of variable *n*. Based on the transmitter and receiver modules of the ZPW-2000A track circuit system, the circuit golden model was designed using Verilog. The test pattern of the golden model was generated, and the fault coverage was up to 100%. The large-scale circuit fault coverage cannot easily reach 100%. The algorithm performance was tested using medium-scale international standard circuits S713 and S1423 and a large-scale international standard circuit S9234. In the same generation time, the fault coverage is improved by more than 50% and 30% as compared with the independent method. The time advantage of the pseudorandom pattern generation algorithm and the coverage advantage of the D algorithm were effectively combined. Therefore, it can be better used in the subsequent construction of high-speed railway equipment test platforms.

## Figures and Tables

**Table 1:** Generated results of each algorithm.

	Number of Patterns Generated			Number of Faults Detected			Fault Coverage		
	S713	S1423	S9234	S713	S1423	S9234	S713	S1423	S9234
Pseudorandom pattern generation	26	68	79	529	1628	6005	58%	65%	53%
D algorithm pattern generation	13	37	71	586	1833	7703	64%	73%	68%
Time-varying pseudorandom disturbed pattern generation	28	125	177	815	2511	10921	89%	98%	96%
